# Iran’s Bushehr Earthquake at a Glance

**DOI:** 10.1371/currents.dis.b69b729791d032b6a1e0f5f9ac4571a4

**Published:** 2013-05-01

**Authors:** Hadi Mohammad Khanli, Mohsen Sokouti, Ata Mahmoodpoor, Kamyar Ghabili, Samad E J Golzari, Amir Mohammad Bazzazi, Alireza Ghaffari, Farzad Nami, Babak Sabermarouf

**Affiliations:** Faculty of Medicine, Tabriz University of Medical Sciences, Tabriz, Iran; Department of Cardiovascular Surgery, Tabriz University of Medical Sciences, Tabriz, Iran; Department of Anesthesiology and Intensive Care Medicine, Tabriz University of Medical Sciences, Tabriz, Iran; Physical Medicine and Rehabilitation Research Center, Tabriz University of Medical Sciences, Tabriz, Iran; Cardiovascular Research Center, Tabriz University of Medical Sciences, Tabriz, Iran; Students’ Research Committee, Tabriz University of Medical Sciences, Tabriz, Iran; Department of Neurosurgery, Urmia University of Medical Sciences, Urmia, Iran; Medical Philosophy and History Research Center, Tabriz University of Medical Sciences, Tabriz, Iran; Physical Medicine and Rehabilitation Research Center, Tabriz University of Medical Sciences, Tabriz, Iran; Neurosciences Research Center (NSRC), Tabriz University of Medical Sciences, Tabriz, Iran

## Abstract

On 9 April 2013, an earthquake of 6.1 magnitude hit southwestern Iran near the city of Khvormuj and the towns of Kaki and Shonbeh in Bushehr province. The official disaster mitigation committee took immediate actions to coordinate rescue teams equipped with 24-hour medical air assistance. Overall, 160 victims were transferred to and treated in the Khvormuj hospital, while 139 survivors were transferred to the hospitals in Bushehr for specialized care. The survivors have been settled in temporary shelters with adequate primary supplies. Considering the hot climate of the area, immediate measures should be taken in order to avoid any further casualties particularly heatstroke, dehydration, diarrheal and vector-borne diseases.

## Brief Incident Report

Less than a year after devastating twin earthquakes in northwestern Iran, an earthquake of 6.1 magnitude hit southwestern Iran near the city of Khvormuj and the towns of Kaki and Shonbeh in Bushehr province on 9 April 2013.^1^ ^2^ ^3^ Being felt in Persian Gulf coast, Qatar, Bahrain, UAE, and eastern Saudi Arabia, the quake was so massive that led to destruction of three thousand houses, 37 deaths (mostly in the town of Shonbeh and its villages) and more than a thousand casualties.^4^ Thanks to the previous experiences from the 2012 twin earthquakes, the official disaster mitigation committee took immediate actions to coordinate rescue operations.^5^ ^6^ Continuous efforts were channeled into searching for the rubbled victims; however, two villages in the Khvormuj district were almost flattened. People rushing from the neighboring areas to offer their help slowed down the rescue process, a similar obstacle faced in 2012 twin earthquakes.^7^ Overall, 160 victims were transferred to and treated in the Khvormuj hospital, a university-affiliated hospital in Khvormuj district that provided abundant medical services obviating any field hospitals establishment. Nevertheless, 139 survivors were transferred to the hospitals in Bushehr for specialized care. Telecommunication was hampered within the early hours after the earthquake resulting in discoordination among the rescue teams. The similar concern was reported in the 2012 earthquakes in northwestern Iran.^5^ ^8^ Except for an emergency need for blood supply which was later met by the neighboring provinces, no serious medical shortage was reported. Unlike the previous experiences in 2012 earthquakes, the number of the fatal casualties was minimal which might have been due to the sufficient 24-hour medical air assistance.^5^

The survivors have been settled in temporary shelters with adequate primary supplies. Moreover, a psychosocial support team has been sent to the field as the aftershocks are still continuing. However, considering the hot climate of the area, immediate measures should be taken in order to avoid any further casualties particularly heatstroke, dehydration, diarrheal and vector-borne diseases.^9^ ^10^ ^11^ ^12^ ^13^

## Funding Statement

None

## Competing Interests

The authors have declared that no competing interests exist.

## Correspondence

Dr. Samad EJ Golzari. Email: drgolzari@hotmail.com
